# RETRACTED: Atta et al. Functionalization of Magnetite Nanoparticles as Oil Spill Collector. *Int. J. Mol. Sci.* 2015, *16*, 6911–6931

**DOI:** 10.3390/ijms27010006

**Published:** 2025-12-19

**Authors:** Ayman M. Atta, Hamad A. Al-Lohedan, Sami A. Al-Hussain

**Affiliations:** 1 Chemistry Department, College of Science, King Saud University, Riyadh 11451, Saudi Arabia; 2 Egyptian Petroleum Research Institute, 1 Ahmad Elzomor St., Nasr City, Cairo 11727, Egypt; 3 Chemistry Department, Faculty of Science, Al-Imam Muhammad Bin Saud Islamic University, Riyadh 11632, Saudi Arabia

The journal retracts the article “Functionalization of Magnetite Nanoparticles as Oil Spill Collector” [1] cited above.

Following publication, concerns were brought to the attention of the Editorial Office regarding the integrity of a number of images presented in this publication [1].

Adhering to our standard procedure, an investigation was conducted by the Editorial Office and Editorial Board that identified indications of inappropriate editing in Figures 1, 2 and 4 and partial duplication in Figure 7B presented in this article [1], with earlier publications [2,3,4] presenting different experimental conditions. Consequently, the Editorial Board has lost confidence in the reliability of the findings and has decided to retract this publication, as per MDPI’s retraction policy (https://www.mdpi.com/ethics#_bookmark30).

This retraction was approved by the Section Editor-in-Chief of the *International Journal of Molecular Sciences*.

The authors did not provide a comment on this decision.
